# Effects on the maxilla and cranial base caused by cervical headgear: A longitudinal study

**DOI:** 10.4317/medoral.17698

**Published:** 2012-05-01

**Authors:** Juan Alió-Sanz, Carmen Iglesias-Conde, José Lorenzo-Pernía, Alejandro Iglesias-Linares, Asunción Mendoza-Mendoza, Enrique Solano-Reina

**Affiliations:** 1 DDS, MS, PhD. Professor of orthodontics, Complutense University of Madrid, Spain; 2 DDS, PhD. Private practice, Ourense, Spai; 3 DDS, PhD. Private practice, Madrid, Spain; 4 DDS, MSc, PhD, Lecturer Masters Programme in Orthodontics and Dentofacial Orthopaedics. School of Dentistry. University of Seville, Spain; 5 DDS , MSc, PhD, Professor of paedriatic dentistry, University of Seville. Spain; 6 DDS, MSc, PhD, Chairman of orthodontics, University of Seville. Spain

## Abstract

Objectives: The aim of this study is to test the possible orthopedic effects of cervical headgear on the cranial base and maxilla. Study design: a sample consisting of 79 subjects with skeletal class II malocclusion was divided into two groups. The experimental group was made up of 41 patients all treated with cervical headgear. The control group included a total of 38 non-treated patients. Each one of these groups was then subdivided according to age into one of three groups: prepubescent, pubescent or post-pubescent. Cephalometric parameters were compared in both groups in order to measure the cranial base angle and the vertical and sagittal position of the maxilla. Additionally, cephalometric superimpositions taken at the beginning and end of the study were compared. Results: results revealed significant differences in the cranial base angle and in the SNA angle (p<0.05). However, no differences were observed in the variables that measure the maxillomandibular relationship. While no changes were noted in the palatal plane slope, a flattening of the cranial base was found caused by the cervical headgear, in addition to a retrusion of point A that does not mean there was a reduction in the maxillomandibular relationship. Conclusions: cervical headgear treatment induces cephalometric flattening of the cranial base and a decrease of the SNA angle.

** Key words:**Orthodontics, cervical headgear, class II treatment, cephalometry, superimposition.

## Introduction

Cervical headgear as a routine treatment for skeletal class II malocclusions has been used since the beginning of the 20th century to date, being a matter of study of numerous studies (1-5). More than 40 years ago, this appliance was proven to have observable effects on the anterior nasal spine and in the rotations of the palatal plane and even on the surrounding craniofacial structures (6) referred to a retrusion of point A and a clockwise rotation of the palatal plane. This effect has been found since then in numerous studies (7).

The force applied by headgear can be cervical, low and occipital or medial (8,9). Medial traction would be applicable to those patients with strong vertical growth. Cervical traction tends to inhibit maxillary growth with a clockwise slope of the palatal plane, while medial traction tends to hold that plane steady (8-10).

Droschl (11) carried out a study on the effects of cervical headgear in monkeys finding a strong clockwise rotation of the maxillary bone along with a retrusion of this bone due to the retrusive force. Later studies corroborate these results (12). Meldrum (13) conducted a similar study but with occipital headgear and found a parallel drop in the palatal plane. Other authors have studied the effects combining different kinds of traction (14) and found that neither the maxillary nor the palatal plane position was significantly affected.

Besides the type of traction, the effects of headgear are influenced by the height of the external rami of the facial arch. Melsen (15) found the greatest retrusive effect on the maxilla occurred when the rami were very high. The orthopedic effect on the maxilla not only refers to the possible stunting of growth in this bone but also to a clear distalizing effect (16).

Nowadays, the main controversy lies in the question of whether to start orthopedic treatment, thereby affecting craniofacial growth, or on the contrary, to choose not to use this kind of treatment and fall back on orthognathic surgery once the patient has reached the appropriate age. There is certain general agreement regarding the therapeutic possibilities of functional apparatus in redirecting growth (17). This also applies to the orthopedic effects of headgear. Nonetheless, even though the studies are quite numerous in this respect, the conclusions are sometimes not applicable to the general population due to the disparity in criteria used in the samples studied, the age of the patients or the very design of the study (18). There is also disparity in criteria in some of the effects described. Some authors, obtain an increase in the angle of the palatal plane with the anterior cranial base (19). Some studies claim that with cervical headgear changes are produced in the pterygomaxillary suture (7,20). Wieslander and Buck (21) found that there was a clockwise slope of the sphenoidal plane. There is complete unanimity about the retrusive effect that cervical headgear exercises over point A (21,22). On the other hand, this unanimity is nonexistent regarding changes in the palatal plane angle. Some authors refer to a posterior rotation of the plane with a greater drop in the posterior nasal spine (8,14). Still other authors do not obtain any slope of the palatal plane (16,23). There is also controversy with respect to the effects on the mandible. Many studies find an increase in the slope of the mandibular plane caused by a clockwise turn of this bone under treatment with cervical headgear (7). Other authors have found the opposite effect, that is, a decrease in the value of this angle (16). Some authors do not find any changes in the slope of the angle of the mandibular plane at all (23).

In order to determine cervical headgear effects on the cranial base angle, on the sagittal position of the upper jaw, and on the potential changes in the palatal slope in different age treatment periods (prepubescent, pubescent or post-pubescent) and how they affect the maxillomandibular relationship, a clinical retrospective study was performed on a representative sample of a Southern European population.

## Material and Methods

-Sample

The sample group used in this study was made up of 79 subjects from the Master’s in Orthodontics Program at the Complutense University of Madrid. The selection criteria of this group were the following: 1) subjects between 8-18 years old; 2) no dental pieces missing; 3) no apparent craniofacial abnormalities; 4) no prior orthodontic treatment; 5) absence of hypodontia and dental abnormalities; 6) cephalometric class II malocclusion with a minimum convexity of 5º and an ANB greater than or equal to 5º (24); 7) Caucasian origins.

The above mentioned group was divided into two different groups: the experimental group and the control group. The separation was carried out based on the willingness of the patients to start treatment, with the control group being those who did not intend to actually begin the treatment. Each group was then subdivided into three subgroups depending on the age of the patient, namely: the prepubescent group, 8-11 years old (n: 36); the pubescent group, 12-14 years old (n:32); and the post-pubescent group, 15-18 years old (n:17).

The control group was made of 38 patients (n: 18 prepubescent, 20 pubescent, 6 post-pubescent). The experimental group was made up of 41 patients (n:18 prepubescent, 12 pubescent,11 post-pubescent), 21 women and 20 men. All of the patients were fitted with a face-bow (GAC, New York) connected to bands on the first upper molars with the face-bow tube placed on the gums. Cervical headgear was given to all the patients exerting a force of 500 g. per side, applied between 12 and 14 hours a day. All subjects were given a check-up once a month and received treatment for at least one year, and the total follow-up period was three years and six months. The average age of this group at the beginning of treatment was 10 years / 2 months and at the end it was 13 years / 8 months. The control group was made up of 38 patients, 16 women and 22 men. The average age at the beginning of the study was 10 years / 2 months and at the end it was 13 years / 8 months.

-Radiographic and cephalometric records

Lateral x-rays were performed on every subject at both the start and end of treatment.

Using computerized analysis, cephalometric records were traced on all lateral cranium x-rays (Nemoceph Studio, Nemotec Dental System) and the following cephalometric reference points were marked: Sella Turcica (S), Basion (Ba), Nasion (N), Point A, Ante-rior Nasal Spine (ANS), Posterior Nasal Spine (PNS), Porion (Po), Pterygomaxillary Suture (Cf), Point C (C), Suborbital (Or), Condylion point (Co), Pogonion (Pg) and Gonion angle (Go). A set of angular parameters was traced over the points mentioned (SNA, ANB, maxillary depth, maxillary height, slope of the palatal plane and cranial deflection) as well as linear parameters (convexity, posterior facial height and distance from the point to the perpendicular to the Nasion and maxillary length) ( Table 1).

**Table 1:**
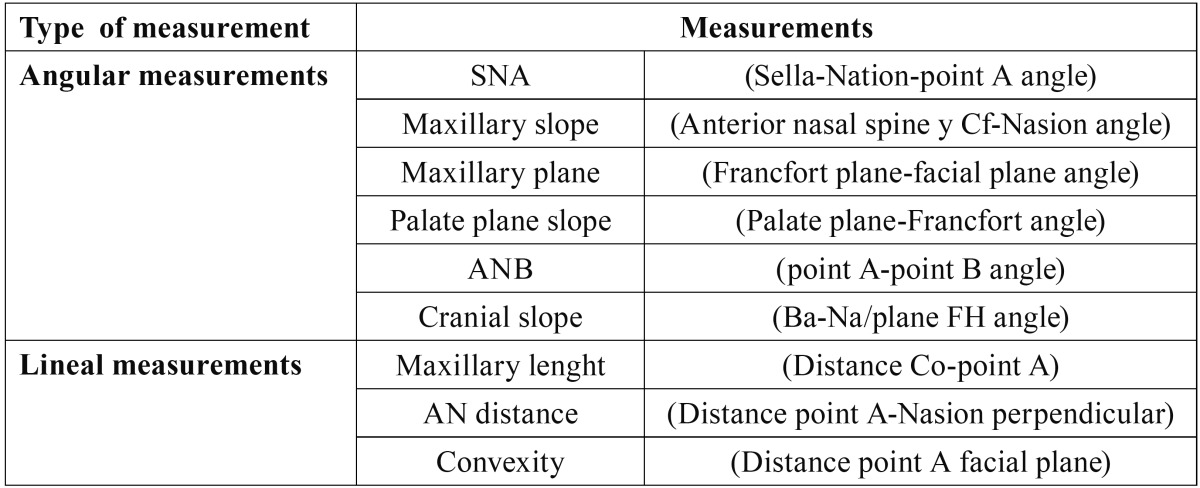
Maxillary parameters.

Superimpositions of the initial and final traces were carried out in order to evaluate how much growth had taken place in the Ba-N plane, using N as the fixed point. Both initial and final point A positions were projected over the Frankfort plane as a horizontal reference. For the vertical reference plane, we projected the anterior and posterior nasal spine positions over the vertical pterygoid in both the initial and final measurements. Positive values were applied when the final point A position was in front of the initial point A position, and similarly, when the final nasal spine position was lower than the initial one. We also took into account any rotations that might have arisen in the palatal plane. A positive rotation was defined as when the final palatal plane position had changed in a counterclockwise direction with respect to the initial position, and vice versa, a negative value was assigned to a clockwise rotation.

-Statistics

All the cephalometries were traced by two experimented researchers (J.A.S. and C.I.C.) belonging to the general research project on growth carried out in the Master’s Program in Orthodontics at the Universidad Complutense of Madrid. These researchers calibrate their measurements annually to avoid any error in the cephalometric tracings. In order to estimate the intra-examiner variation for the radiological evaluation all the radiographs were evaluated twice by the same experienced examiner (J.A.S.). In order to estimate the inter-examiner variation all the radiographs were evaluated by a second experienced examiner (C.I.C.). Once both researchers have performed the tracings, they were compared to each other and results analysed as previously described (25).

A descriptive statistical analysis was used to evaluate the data obtained in which the arithmetic mean, standard deviation, per-centiles and rank (maximum and minimum values) of each variable by sex and age group were included. Later, an analytical or inferential statistical analysis was done. In order to study the evolution of each variable over time and establish comparisons in the behavior shown by any one variable in each age group, an analysis of variance (ANOVA) followed by Duncan’s multiple range test was used as a test a posteriori with a reliability of 0.05, after verifying for normal distribution with a Q-Q plot and for homogeneity of variance (p>0.05 at Levene´s test). A Student t for independent samples was applied to study the differences in function of sex, after verifying for randomness (the Wald-Wolfowitz runs test at p>0.05 for all variables in the two groups) and for normality (p>0.05 at Shapiro–Wilks test).

## Results

In  Table 2are shown the results of the eight cephalometric variables in the total sample and in each one of the age groups (prepubescent, pubescent and post-pubescent) for the experimental group (EG) as well as for the control group (CG). Significant differences were found in the cranial base angulation in the three age groups and in the total sample ( Table 2). We also found very significant differences (p<0.001) in the SNA angle in all of the groups except the post-pubescent group ( Table 2). There were differences regarding maxillary height in the pubescent group, which was the group that had the greatest number of patients undergoing treatment ( Table 2). Maxillary depth (Maxillary D.), as in the case of the SNA angle, showed significant differences in all the groups except in the oldest ( Table 2). The slope of the palatal plane (S. palatal), however, did not show any significant differences in any of the groups. In addition, it is interesting to note that the ANB angle did not show any significant differences in any age group or in the total sample. As for linear measurements, maxillary length only showed significant differences in the pubescent group. However, the distance from point A to the facial plane was only significant in the total sample, but in none of the age groups. Convexity, as in the case of the ANB angle, did not show any significant differences in any of the age groups or in the total sample ( Table 2).

**Table 2:**
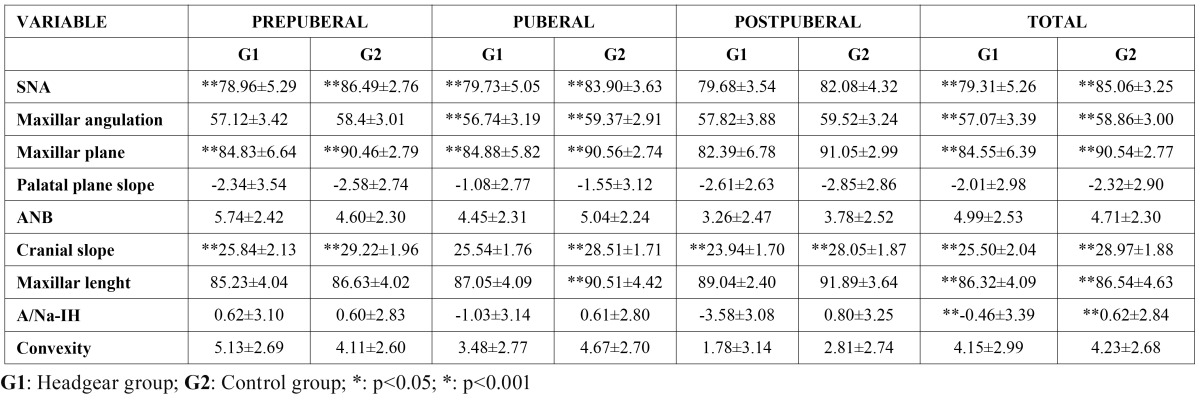
Maxillary cephalometric variables distributed by gropusof age. Treatment group (G1) and control group (G2).

In the total sample, the only variable that was significantly different between the two groups, comparing the superimpositions of the initial and final measurements, was the one having to do with point A, showing a displacement towards the back in the treatment group ( Table 3). This result was seen in all three age groups. Significant differences were also seen in the prepubescent group regarding changes in the anterior nasal spine which was observed to have undergone more descent in the experimental group. None of the other superimposition points showed significant differences in the total sample or in the groups arranged according to age ( Table 3).

**Table 3:**
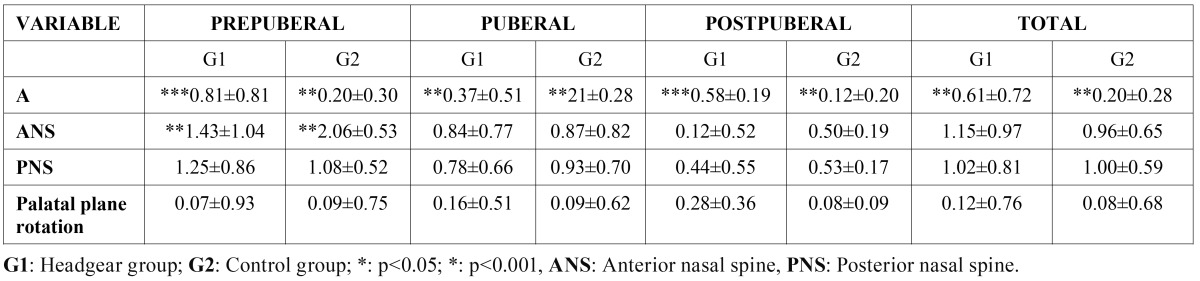
Superimpositions.

According to sex, significant differences resulted in the SNA angle value between men and women in both the control group as well as the experimental group ( Table 4). We have also found significant differences in the maxillary depth in the experimental group, but not in the control group ( Table 4). The effective maxillary length showed significant differences between the sexes in both groups. Differences were not found between the sexes in the rest of the parameters. ( Table 4) However, no statistically significant differences between sexes were found in relation to the cephalometric superimpositions.

**Table 4:**
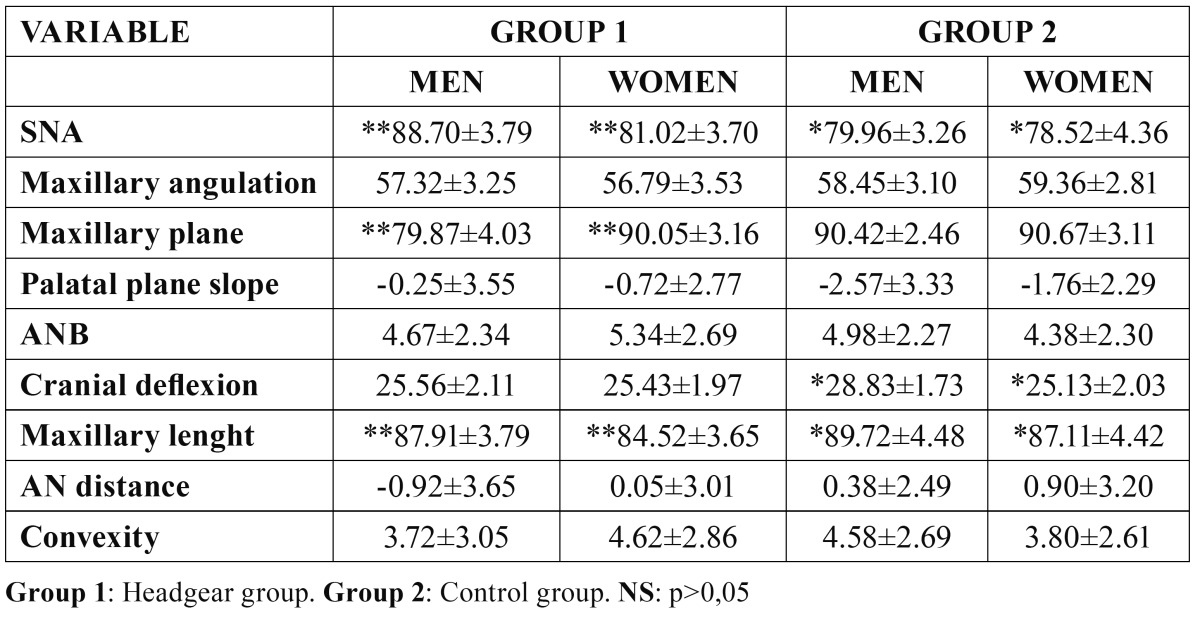
Cephalometric variables distributed by gender.

## Discussion

-Cranial base 

The variable related to the cranial base evaluated in this study is the angle of cranial deflection. This angle showed similar behavior in the two groups. However, an important point to bear in mind is the significant difference that appears between the two groups, both in the assessment of the total sample as well as in that of all three age groups. This suggests that the plane of the cranial base (Ba-Na) is flatter in the treatment group as if the headgear caused a clockwise rotation in this plane, with a lowering of the nasion point.

Some studies (19) have demonstrated the effects of headgear on the cranial base due to the clockwise rotation this undergoes and to an increase in SN length. Other studies find a remodeling of the pterygomaxillary suture with affectation of the sphenoid bone caused by the clockwise rotation brought about by headgear (7).

-Maxillary sagittal changes

In order to assess sagittal maxillary changes a series of angular (SNA, maxillary depth and ANB angle) and linear (maxillary length, distance from point A to the perpendicular from the nasion and convexity) measurements were used. By means of the cephalometric superimposition system the change of point A in the sagittal plane was assessed. The results show that there is an important retrusive effect on the maxilla in the experimental group with headgear. Similar effects have been noted by other authors (7,26). It is worth considering whether the decrease in the SNA angle is due to a restrictive effect on the maxilla, to a reabsorption in the point A area caused by the distal movement of the front teeth (27), or to an increase in cranial length.

We did not obtain significant differences in the SNA angle in the oldest group, most likely because the orthopedic effect in these patients is very limited. Therefore, the use of headgear with orthopedic ends would not be recommended for patients over 15 years of age. In this respect, some authors advocate starting treatment at the end of pubescence (26). Along these lines, our results show a sagittal orthopedic effect of equal magnitude in the youngest patients (prepubescent group) compared to the patients belonging to the pubescent group. Other authors advocate starting treatment with headgear in the prepubescent period because during this period the orthopedic effect is greater (21). When examining the effective maxillary length results we see that there is a significant decrease in this variable but only in the pubescent group. When studying the superimpositions of point A in the three groups ( Table 3) we observe that significant differences are found in all of them. All of this leads us to believe that the retrusive effect occurs in any age group and that this fact can be compensated for by the effect brought about in other structures of the craniofacial complex. Our results reveal a greater retrusive effect in women even though this is only seen in the SNA value and not in the point A superimposition measurements.

The decrease in angle values that place the maxilla in the anteroposterior plane does not go hand in hand with a decrease in the values that measure the maxillomandibular relationship, that is, the ANB angle and convexity. Even though mandibular parameters were not measured in this study, everything seems to indicate that the reason for this behavior is the effect of mandibular postero-rotation, which is a result of using cervical headgear. Other authors (20,27) actually have found a decrease in the ANB angle along with the SNA. However, if we look at the figures of each one of these variables and their evolution over time (Figures 4 and 5) we can see that a continuous, constant decrease in value is produced in both cases even though this decrease may not be statistically significant. Mandibular postero-rotation might be greater if the patient has a vertical growth pattern. Haralabakis et al. (28) found a similar decrease in this angle in patients with both a high as well as with a low mandibular plane angle. Other studies have obtained similar results (23).

-Vertical maxillary changes

Vertical changes in the maxilla were measured using the angle of maxillary height and the slope of the palatal plane. Using cephalometric superimpositions, we found that there were significant differences in maxillary height in the total sample and in the pubescent group. The value of this angle is less than in the group treated with headgear. The reason for this result seems to be that it stems from the flattening which occurs in the cranial base with the lowering of the nasion. The angle would not change if the maxilla rotated clockwise at the same time as the cranial base flattened. Therefore, we must assume that the lowering of the nasion is greater in magnitude that the possible clockwise rotation of the palatal plane. This fact is supported by the lack of significant differences in the value of the slope of the palatal plane. Some authors obtain different results with a clockwise slope of this plane (9). Other studies do not obtain differences in the slope of this plane in those patients treated with headgear (27). Braun et al. (29) found a greater slope of the palatal plane in the group of patients treated with headgear, with this slope being even greater in male patients. We did not find any differences between the two sexes.

The anterior nasal spine moves down more in the group treated with headgear ( Table 3), nonetheless, these differences were only statistically significant in the prepubescent group. Even in the first studies done with headgear (1) a lowering of the ANS due to a clockwise rotation of the palatal plane was found in patients undergoing treatment. Boecler et al. (23) found a greater lowering of the ANS in patients treated with headgear, however, no significant differences appeared in his group of untreated patients. It would seem that this point is much less affected by headgear than other points of reference and that it is only possible to alter it sufficiently at an early age.

The posterior nasal spine did not show any significant differences in any of the age groups. Therefore, this point is stable and does not vary with the use of headgear. Lima et al. (26) have obtained similar results claiming that, due to the headgear being inserted at the molar level, the retrusive force is greater at the PNS level in comparison to the ANS so that the stunting of normal maxillary growth will be greater at this posterior point. Some authors (7,8) found a more posterior position of the PNS in treated patients. According to these authors, this could be due to the effect that using cervical headgear can have on adjacent bone structures.

From the above it may be deduced that the slope of the palatal plane will not have significant differences between the two groups. This important point suggests that the clockwise rotation of the maxilla with cervical headgear is not such a conclusive fact as has often been affirmed and that the effect of headgear is stronger in the cranial base than in the maxilla with respect to vertical changes.

Some authors (28) claim that the possibility of affecting the slope of the palatal plane with cervical headgear in dolichofacial patients is greater than in brachyfacial patients. Other studies point out the relationship between the type of traction (low or middle) and the effects on the palatal plane. Many authors obtain a greater slope with low traction (6). However, other studies (9,23) do not obtain any differences between the two types of traction, with no repercussion at the profile esthetics (30).

## Conclusions

1. All three age groups showed a statistically significant flattening of the cranial base when treated with headgear. This effect on the cranial base is the most evident of all those found.

2. There is an important retrusive effect on the maxilla in the prepubescent and pubescent groups. This effect is directly related to the more posterior position of point A after treatment.

3. Despite the retrusion of point A, a decrease in the maxillomandibular relationship did not appear in patients treated with headgear.

4. No rotation effect of the palatal plane was observed in patients treated with headgear.
